# Supplementary material for: The use of a patch to augment rotator cuff surgery – A survey of UK shoulder and elbow surgeons
Source: PLoS One. 2020 Apr 2;15(4):e0230235. doi: 10.1371/journal.pone.0230235 (PMC7117708; doi:10.1371/journal.pone.0230235)
Supplement: S1 Survey — (PDF) [file pone.0230235.s001.pdf]

PARCS | PATCH AUGMENTED ROTATOR CUFF SURGERY

PARCS Study Survey (BESS Membership)

Introduction

A survey of current practice and opinions on using patches to enhance rotator cuff surgery.

The aim of this survey is to get BESS members' views on the use a patch to augment Rotator Cuff Surgery, and to explore current practice. It is part of a larger study (called the PARCS feasibility study) which is looking at the feasibility and design of a randomised trial to assess the clinical and cost effectiveness of patch augmented surgery in the NHS. Studies, such as the UKUFF trial, have indicated a failure rate of up to 40% for cuff repairs. There is growing interest amongst surgeons in the use of patches to provide a support structure or "scaffold" for the repair, to improve tendon fixing to the bone and healing. Various patches have been developed using different materials (e.g. animal heart or synthetic materials), processes and sizes. It is unclear to what extent they have been used in the NHS, which patients are best suited to patch augment, and the degree of support amongst surgeons for a randomised trial.

This survey addresses these uncertainties and will help inform future trials in this area of research. Findings will be circulated to BESS membership, presented at a BESS meeting and published in an academic journal.

We would be extremely grateful if you could take the time (approximately 10 minutes) to complete this short survey.

About you

What is your position? ✱ *Required*

Please select exactly 1 answer(s).

- [ ] Consultant Shoulder Surgeon
- [ ] Orthopaedic Trainee
- [ ] Other

Other, please state:

About you

Where do you work? ✱ *Required*

Please select between 1 and 4 answers.

- ☐ District General Hospital
- ☐ University/ Teaching Hospital
- ☐ Private Hospital
- ☐ Other

Other, please state:

About you

What is your surgical practice regarding rotator cuff repair? ✱ *Required*

Use of a patch to augment rotator cuff surgery

Have you used a patch to augment rotator cuff surgery? ✱ *Required*

Use of a patch to augment rotator cuff surgery

How many rotator cuff operations have you performed using a patch? ✱ *Required*

Please enter a whole number (integer).

The number should be 1 or greater.

Which patches have you used? ✱ *Required*

Why did you use these specific patches? ✱ *Required*

Please use this space to add any further comments relating to choice of patch for using to augment rotator cuff repair.

Patinet suitability for a patch augmented rotator cuff repair

It is unclear in which patients patch augmented surgery would be considered appropriate. We would like to know your views on when patch augmentation is, or might be, suitable.

Age and tear size have been suggested to be the two main factors affecting outcome after rotator cuff repair surgery. The table below shows 16 combinations of age and tear size. If you think a patient with a particular combination would be suitable please choose 'yes'. If you would never consider such a patient to be suitable then choose 'no'. If you are unsure about whether they would be suitable choose 'unsure'.

In the example below patients with small tears are never considered suitable whereas those with large and massive tears always are. The suitability of those with a medium sized tear in uncertain. Age did not affect the decision for any size of tear.

Tear Size/ Patient Age	Yes	No	Unsure
50 year old, small		X	
60 year old, small		X	
70 year old, small		X	
80 year old, small		X	
50 year old, medium			X
60 year old, medium			X
70 year old, medium			X
80 year old, medium			X
50 year old, large	X		
60 year old, large	X		
70 year old, large	X		
80 year old, large	X		
50 year old, massive	X		
60 year old, massive	X		
70 year old, massive	X		
80 year old, massive	X		

Your response

Please don't select more than 1 answer(s) per row.

Please select exactly 16 answer(s).

	Yes	No	Unsure
50 year old, small	☐	☐	☐
60 year old, small	☐	☐	☐

70 year old, small	☐	☐	☐
80 year old, small	☐	☐	☐
50 year old, medium	☐	☐	☐
60 year old, medium	☐	☐	☐
70 year old, medium	☐	☐	☐
80 year old, medium	☐	☐	☐
50 year old, large	☐	☐	☐
60 year old, large	☐	☐	☐
70 year old, large	☐	☐	☐
80 year old, large	☐	☐	☐
50 year old, massive	☐	☐	☐
60 year old, massive	☐	☐	☐
70 year old, massive	☐	☐	☐
80 year old, massive	☐	☐	☐

Please feel free to use this space to add any further comments relating to suitability of a patient for a patch augmented rotator cuff repair.

Interest in participating in a randomised trial

Would you be interested in taking part in a randomised controlled trial of patch augmented surgery? (i.e. your patients being approached to take part, and you would perform the study operations, etc.) ✱ *Required*

- Yes
- Maybe
- No

Interest in participating in a randomised trial

Is there anything that would make you more interested in taking part in such a study?
* *Required*

- ○ Yes
- ○ No

Yes, please give details:

Final Questions

Please feel free to use this space to comment on any aspect related to this topic or survey that you feel is important.

Final Questions

Would you be happy for us to contact you if we have further questions? ✱ *Required*

- Yes
- No

If Yes, please provide your e-mail address

Please enter a valid email address.

Thank you

THANK YOU FOR TAKING THE TIME TO COMPLETE THIS SURVEY!

YOUR HELP IS GREATLY APPRECIATED.

If you have any queries about this survey or about the PARCS Study please contact: Andrew Carr on (01865) 223404 or at rcrstudies@ndorms.ox.ac.uk

---

Key for selection options

3 - What is your surgical practice regarding rotator cuff repair?
- All/ predominantly arthroscopic rotator cuff repair
- All/ predominantly open rotator cuff repair
- Substantial amount of both arthroscopic and open repairs

4 - Have you used a patch to augment rotator cuff surgery?
- No
- Yes, in the last 6 months
- Yes, but not in the last 6 months
