# Supplementary material for: The use of a patch to augment rotator cuff surgery – A survey of UK shoulder and elbow surgeons
Source: PLoS One. 2020 Apr 2;15(4):e0230235. doi: 10.1371/journal.pone.0230235 (PMC7117708; doi:10.1371/journal.pone.0230235)
Supplement: Attachment — Submitted filename: Review comments.docx [file pone.0230235.s003.docx]

Patch has been increasingly used in rotator cuff repair, especially for large/massive rotator cuff tear. But there are limited epidemiologic studies about the choice of patch. This paper focused to survey the current UK practice and opinion relating to the factors that influence choice of path, current patient selection and willingness to assist with generation of improved evidence. This study was well performed and provided the panorama of patch uses in the UK. Here are some questions for the authors.

1. Why the authors choose the 6 months as one time point in the survey contents?

2. The date was collected from different surgeons. So please clarify how to validate these date.

3. Why did you preclude the ages less than 50 in the survey contents?

4. Arthroscopic rotator cuff repairs accounted for 66%, but this was not according with the data in Table 2.

5. Clarify the meaning of “Number of patches implanted” in Table 2. Does that mean the number of patch used in one surgery or the total number of patch used by one surgeon?
